# Pyosalpinx due to *Cronobacter sakazakii* in an elderly woman

**DOI:** 10.1186/s12905-021-01283-8

**Published:** 2021-04-01

**Authors:** Satoshi Ohira, Eri Ikeda, Kyosuke Kamijo, Tomokuni Nagai, Koji Tsunemi, Natsuki Uchiyama, Naoki Matsubara, Ryota Tachibana

**Affiliations:** Department of Obstetrics and Gynecology, Iida Municipal Hospital, 438 Yawatamachi, Iida, 395-8502 Japan

**Keywords:** *Cronobacter sakazakii*, Pyosalpinx, Adult, Elderly woman, Case report

## Abstract

**Background:**

*Cronobacter sakazakii* (*C. sakazakii*) is a bacterium known to cause severe neonatal infections in premature infants with the consumption of contaminated powdered milk formula. Adult infections are rare, and there have been no reports of pyosalpinx due to *C. sakazakii* to date.

**Case presentation:**

We report a case of left pyosalpinx due to *C. sakazakii* in a sexually inactive postmenopausal woman. A 70-year-old woman presented to our hospital with left lower abdominal pain and fever. Abdominal computed tomography disclosed a cystic mass continuous with the left edge of the uterus. Urgent laparotomy revealed a ruptured left pyosalpinx with pus-like content. Left salpingo-oophorectomy, resection of the right tube, and washing of the abdominal cavity with saline were performed. Pathological examination of the left adnexa showed tubal tissue with acute inflammation and inflammatory exudate, which were compatible with pyosalpinx, and pus culture yielded *C. sakazakii*.

**Conclusions:**

This is the first case report of pyosalpinx due to *C. sakazakii*. *Cronobacter sakazakii* infections in adult women might occur in the elderly, whose immunity has weakened. Further accumulation of cases of *C. sakazakii* infection is needed to clarify the etiology and behavior of *C. sakazakii* in adults.

## Background

*Cronobacter sakazakii* (*C. sakazakii*) is a Gram-negative, rod-shaped bacterium known to cause severe neonatal meningitis and necrotizing enterocolitis in premature infants with the consumption of contaminated powdered milk formula [1, 2]. Adult infections are rare [1], and there have been no reports of pyosalpinx due to *C. sakazakii* to date. We describe a peculiar case of left pyosalpinx due to *C. sakazakii* in a sexually inactive elderly woman.

## Case presentation

**Fig. 1:**
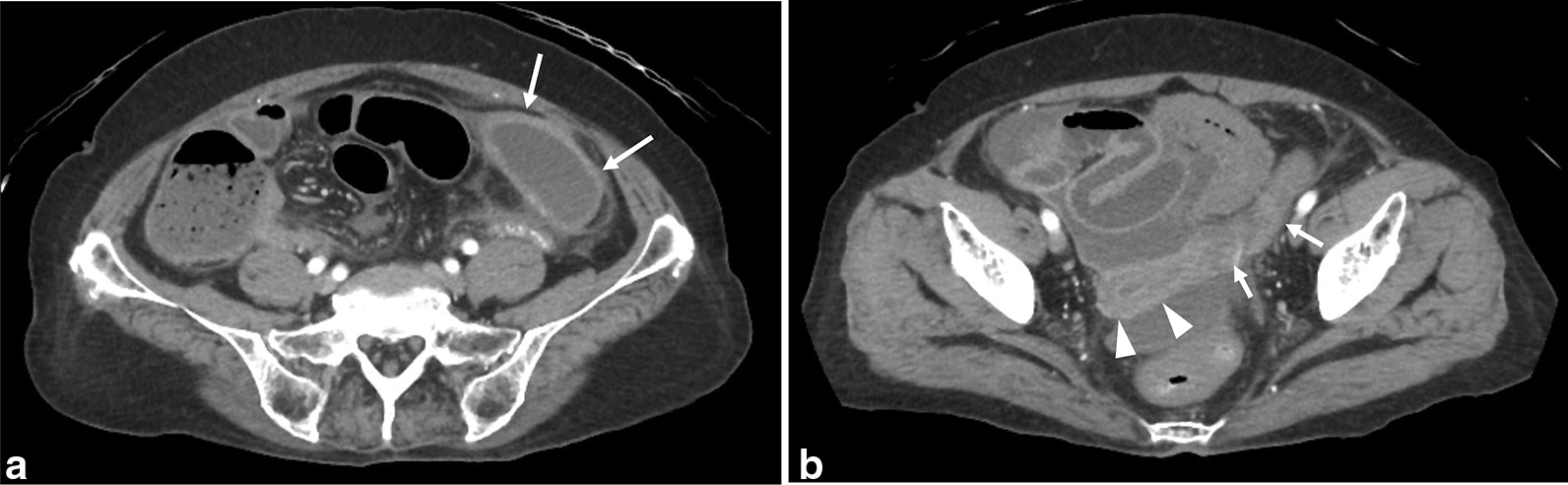
Enhanced abdominal computed tomography demonstrates a cystic mass of 64 × 30 mm with an equally enhanced wall in the left pelvic cavity (panel **a**, arrows). The cystic mass is continuous with the left edge of the uterus (panel **b**, Arrowheads show the uterus, and arrows show a cord structure connected to the mass)

A 70-year-old nulligravid Japanese woman presented to our hospital with a four-day history of left lower abdominal pain and fever up to 38.5 ℃. Three days previously, pyelonephritis had been suspected and an antibiotic was administered at a previous clinic, but her symptoms did not improve. Tracing back her history, she had been totally blind since 14 years old, and diagnosed with depression one year ago and received medication of 15 mg/day of Mirtazapine and 50 mg/day of Chlorpromazine hydrochloride. Her dietary habits were common, but the toothbrushing was insufficient. Therefore, several teeth had been already lost. Her husband had died and she had been sexually inactive for the last four years. Physical examination revealed evident lower abdominal tenderness and rebound tenderness. Her body temperature was 39.4 ℃ and consciousness was drowsy. On gynecological examination, uterine cervical os was tightly closed without vaginal discharge. The uterine body was atrophic. A laboratory test showed leukocytosis with C-reactive protein of 24.36 mg/dL. *Neisseria gonorrhoeae* deoxyribonucleic acid (DNA) and *Chlamydia trachomatis* DNA in the urine were both negative. Urgent abdominal computed tomography (CT) disclosed a cystic mass of 64 × 30 mm with an equally enhanced wall (Fig. 1a). Because the cystic mass was continuous with the left edge of the uterus, a left adnexal abscess was suspected (Fig. 1b). CT also revealed ascites in the pelvic cavity and a diffusely enhanced peritoneum. Diffuse generalized peritonitis caused by rupture of the left adnexal abscess was suspected, and thus surgical intervention was planned.

**Fig. 2:**
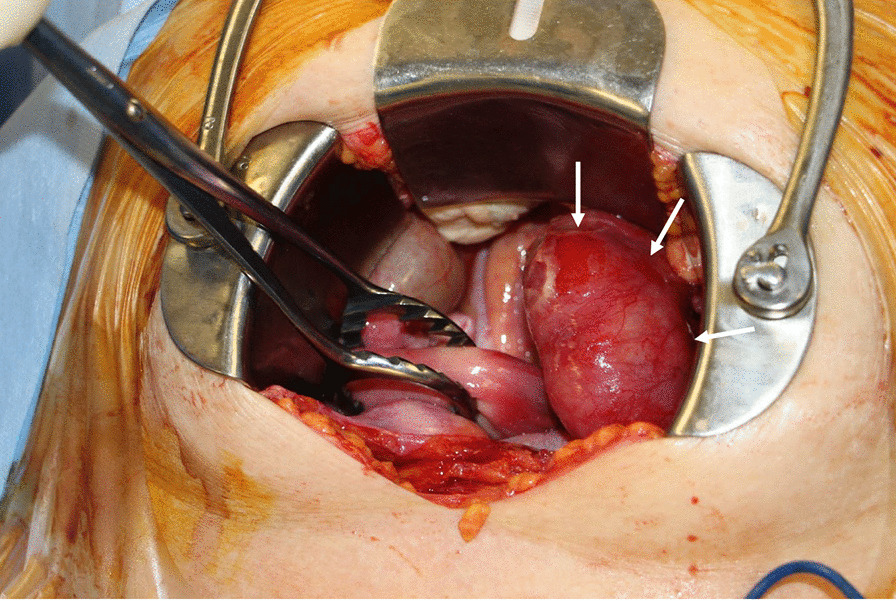
Laparotomy reveals a ruptured left pyosalpinx with pus-like content (arrows)

Laparotomy revealed a ruptured left pyosalpinx with pus-like content (Fig. 2). The ascites had a foul smell and white moss was diffusely attached to the surface of the colon. The left ovary, uterus, and right ovary were atrophic. The right tube was slightly reddish with spreading of inflammation. Left salpingo-oophorectomy, resection of the right tube, and washing of the abdominal cavity with saline were performed. Pathological examination of the left adnexa showed tubal tissue with acute inflammation and inflammatory exudate, which were compatible with a pyosalpinx, and pus culture yielded *C. sakazakii*. In susceptibility testing of antibiotics, although this isolated *C. sakazakii* was resistant to Ampicillin, it was susceptible to other antibiotics including Cefazolin, Cefmetazole, Gentamicin, Levofloxacin, Imipenem, and Meropenem.

Because CO_2_ narcosis developed in the patient, after the operation, respirator management was needed in an intensive care unit. Moreover, polymyxin B-immobilized fiber column direct hemoperfusion was performed for two days. Bacteria were not detected by blood culture performed before the operation, but antibiotics of Cefmetazole sodium for 3 days and Meropenem hydrate for 9 days were administered. The general state of the patient gradually improved and she was discharged from our hospital 21 days after the operation.

## Discussion and conclusions

In 2007, organisms previously classified as *Enterobacter sakazakii* were reassigned to the new genus *Cronobacter* [2]. *Cronobacter sakazakii* has been isolated from clinical sources such as cerebrospinal fluid, blood and sputum, and food such as cheese, meat, and vegetables [2, 3]. Most reported cases of illness caused by *C. sakazakii* involve infants younger than 2 months old [4]. Premature infants with underlying medical conditions are at the greatest risk. Numerous outbreaks caused by *C. sakazakii* have been traced to contaminated powdered infant formula [2]. Meanwhile, 17 cases of illness in adults caused by *C. sakazakii* have been reported in detail [1, 5–13].

Only 7 case reports of *C. sakazakii* infections involving adult women have been published in the literature (Table 1) [1, 5–8]. All of them and the current case were of an advanced age (> 60 years), and 5 cases had underlying diseases such as malignant tumor, atrial fibrillation, cerebral stroke, cecal volvulus, and chronic renal failure. Therefore, it is suggested that *C. sakazakii* infections readily occur in women with weakened immunity. Isolation sites of *C. sakazakii* were blood, sputum, urine and bile. Most patients received surgery and/or the administration of antibiotics, but 4 died. In those cases of *C. sakazakii* infections who subsequently survived, cephem, quinolone or carbapenem antibiotic were administered [1, 5]. The antibiotic therapy including cephem, quinolone and carbapenem might be necessary, but the administration of Ampicillin is not recommended. There have been no reports of pyosalpinx due to *C. sakazakii* to date; therefore, this is the first report of pyosalpinx.

**Table 1:** Case reports of *Cronobacter sakazakii* infection in adult women

References	Age	Isolation site	Clinical presentation	Underlying condition	Treatment (surgery and/or antibiotics)	Outcome
Hawkins et al. [5]	75	Blood	Bacteremia	Atrial fibrillation Cerebral stroke	Cefuroxime, Ceftriaxone Ciprofloxacin	Recovered
See et al. [1]	75	Blood	Splenic abscess	None	Aspiration of abscess Ceftriaxone, Metronidazole Imipenem, Ciprofloxacin	Recovered
Bhat et al. [6]	63	Urine	Urinary tract infection	Chronic renal failure	NA	Died
Lai [7]	73	Bile, Blood	Biliary sepsis	Klatskin tumor	Resection of common bile duct Piperacillin–tazobactam Gentamicin, Imipenem	Died
	82	Blood	Abdominal aortic aneurysm	NA	Aneurysmal repair Ofloxacin, Piperacillin, Tazobactam	Died
	76	Sputum	Pneumonia	Cecal volvulus	Resection of cecum Tobramycin, Ceftazidime, Ofloxacin	Died*
Tsai et al. [8]	64	Sputum	Pneumonia	Breast carcinoma	NA	Recovered
Current case	70	Pus of pyosalpinx	Left pyosalpinx	Depression	Salpingo–oophorectomy Cefmetazole, Meropenem	Recovered

*NA* not available

^*^Pneumonia with isolation of *Staphylococcus aureus* and *C. sakazakii*. *Cronobacter sakazakii* might not be the causative agent of infection

Pyosalpinx and tubo-ovarian abscess are almost always complications of pelvic inflammatory disease and are sexually transmitted infections in many cases. Therefore, pyosalpinx and tubo-ovarian abscess are usually observed in young women; they are rarely found in older women. One hundred and ninety-four cases of pyosalpinx or tubo-ovarian abscess in postmenopausal woman have been reported in the literature [14–17]. Although typical organisms isolated in young women are *Neisseria gonorrhea* and *Chlamydia trachomatis*, those in reported postmenopausal women are *Escherichia coli*, *Clostridium perfringens*, *Peptostreptcoccus*, Group C *Streptococcus*, and *Bacteroides fragilis* [14, 15]. Among physiological mechanisms causing pyosalpinx or tubo-ovarian abscess, ascending infection from the lower genital tract is the most common reason, but ascending infection might be unlikely in sexually inactive postmenopausal women. Another hypothesis of bacterial spread by hematogenous seeding has been proposed as the origin of pyosalpinx in virgin patients [18], and this hypothesis might also be applicable to postmenopausal women. Although the cause of pyosalpinx in our patient remains unclear, we speculate that hematogenous infection by *C. sakazakii* occurred in the hydrosalpinx that existed, and pyosalpinx developed. One of the candidate original infection sites might be the oral cavity [9].

In summary, we describe the first reported case of pyosalpinx due to *C. sakazakii* in a postmenopausal elderly woman. *Cronobacter sakazakii* infections in adult women might be caused to the elderly women whom the immunity decreased. Further accumulation of cases of *C. sakazakii* infection is needed to clarify the etiology and behavior of *C. sakazakii* in adults.

## Data Availability

All data presented in this report are included in this article.

## Abbreviations

*C. Sakazakii*: *Cronobacter sakazakii*
CT: Computed tomography
DNA: Deoxyribonucleic acid
